# A Single Image Deep Learning Approach to Restoration of Corrupted Landsat-7 Satellite Images

**DOI:** 10.3390/s22239273

**Published:** 2022-11-28

**Authors:** Anna Petrovskaia, Raghavendra Jana, Ivan Oseledets

**Affiliations:** 1 Center for Artificial Intelligence Technology, Skolkovo Institute of Science and Technology, 121205 Moscow, Russia; 2 Center for Agro Technologies, Skolkovo Institute of Science and Technology, 121205 Moscow, Russia; 3 Marchuk Institute of Numerical Mathematics, Russian Academy of Science, 119991 Moscow, Russia

**Keywords:** remote sensing, deep learning, single-image approach, deep image prior, Landsat-7

## Abstract

Remote sensing is increasingly recognized as a convenient tool with a wide variety of uses in agriculture. Landsat-7 has supplied multi-spectral imagery of the Earth’s surface for more than 4 years and has become an important data source for a large number of research and policy-making initiatives. Unfortunately, a scan line corrector (SLC) on Landsat-7 broke down in May 2003, which caused the loss of up to 22 percent of any given scene. We present a single-image approach based on leveraging the abilities of the deep image prior method to fill in gaps using only the corrupt image. We test the ability of deep image prior to reconstruct remote sensing scenes with different levels of corruption in them. Additionally, we compare the performance of our approach with the performance of classical single-image gap-filling methods. We demonstrate a quantitative advantage of the proposed approach compared with classical gap-filling methods. The lowest-performing restoration made by the deep image prior approach reaches 0.812 in $r^{2}$, while the best value for the classical approaches is 0.685. We also present the robustness of deep image prior in comparing the influence of the number of corrupted pixels on the restoration results. The usage of this approach could expand the possibilities for a wide variety of agricultural studies and applications.

## 1. Introduction

Remote sensing has become a convenient tool in agriculture, offering researchers and policymakers a wealth of information about the Earth’s surface. Landsat-7 satellites have been supplying multi-spectral imagery of the planet’s surface for over 4 years and become an important data source for a large number of agricultural research and policy-making initiatives [1,2,3,4,5,6]. Unfortunately, a scan line corrector (SLC), which compensates for the forward motion of the satellite, broke down in May 2003 [7]. Since the failure of the SLC, Landsat-7 scenes have exhibited wedge-shaped swaths of missing data that reach 14 pixels in width near the edges. It is estimated that up to 22 percent of any given scene could be lost because of the SLC’s failure, which makes a significant number of Landsat imagery unsuitable for further use in research devoted to any agricultural needs.

A wide variety of gap-filling approaches are reported in the literature to address the issue of missing pixels. All the proposed approaches can be broadly divided into three groups: multi-image, single-image and deep learning methods. Multi-image methods are used to restore damaged Landsat-7 images by utilizing undamaged images of the same area taken before the breakdown of the SLC or by adding other satellites’ products. Numerous geostatistical approaches have been tried to enable inference of the missing pixels. The most well-known geostatistical techniques for tackling this problem are kriging and co-kriging [8,9,10]. In addition, a neighborhood similar pixel interpolator (NSPI) was developed to fill the gaps in SLC-off images [11,12]. The NSPI enables restoring the satellite images of highly heterogeneous landscapes. The authors of [13] provided a new approach called the geostatistical neighborhood similar pixel interpolator, which enhanced the NSPI results. Among the geostatistical methods, the high accuracy in multi-temporal gap filling was also seen by weighted linear regression [14], direct sampling [15] and localized linear histogram matching [12,16]. Generally, using non-corrupted imagery to fill in the gaps has been shown to be more accurate than the single-image approaches. However, the performance of the multi-image methods is inherently dependent on the interval between the acquisition of the two images, where the greater the interval, the lower the reconstruction accuracy, especially in fast-changing conditions such as agricultural regions. However, in many cases, it may not be possible to find a non-corrupted image within a time frame suitable for high-accuracy reconstruction. This is especially important for regions with a high degree of cloud cover and few clear days.

Single-image algorithms primarily rely on the within-image pixel similarities rules, in which non-corrupted areas are used to reconstruct pixels in gaps. Classical geostatistical methods are also usually applied for single-image restoration tasks [17]. Aside from that, a number of authors have recognized methods of low-rank matrix approximations as effective instruments for satellite product restoration. To solve the problem of matrix completion, it is formulated as a low-rank minimization problem approach. Hu et al. [18] applied a matrix completion algorithm by minimizing the truncated nuclear norm. Miao et al. [19] used the low-rank approximate regularization method of dictionary learning. El Fellah et al. [20] proposed a concept based on matrix completion and restoration of the structure’s edges of damaged areas.

Deep learning (DL) methods have been applied to recover satellite images by a significant number of authors. Deep neural networks have a remarkable ability to restore textures based on previously seen examples. For Landsat-7 corrupted products, the most popular DL methods are convolutional neural networks [21,22]. Deep dictionary learning [23] joins the method of dictionary learning with a deep learning approach for better multispectral image inpainting. However, the vast majority of classical deep learning methods require a huge amount of training data to assure effective convergence of the model parameters. Convolutional conditional neural processes (ConvCNPs) and convolutional latent neural processes (ConvLNPs) have been shown to exhibit very good few-shot and zero-shot learning capabilities [24], but these methods still need pretrained models.

To combine the advantages of the single-image and deep learning methods, we propose the usage of deep image prior (DIP) [25] for Landsat-7 image restoration. This novel technique for training deep learning models allows users to perform satellite image restoration without any additional data. The DIP method was previously applied for the inpainting and denoising of hyperspectral images [26,27,28]. However, no studies have been devoted to restoring multispectral images with DIP, which is an important question to be addressed, as multispectral imagery provides significantly less information than hyperspectral imagery. In this paper, we apply a single-image approach based on leveraging deep image prior (DIP)’s ability to fill in Landsat-7 image gaps using only the corrupt image.

The main contributions of this paper are the following:

- We propose an application of the DIP method for the reconstruction task of corrupted Landsat-7 images;
- We demonstrate the ability of DIP to reconstruct remote sensing scenes with different levels of corruption in them;
- We compare the performance of our approach with the performance of classical single-image gap-filling methods.

## 2. Materials and Methods

### 2.1. Deep Image Prior

“Deep image prior” (DIP) [25] is an approach for training convolutional neural networks (CNNs) [29] with image data. The DIP methodology eliminates the need for a pretrained network or an image database. Only the corrupted image (designated as $x_{0}$) is used in the restoration process.

The DIP method is based on the assumption that the image prior can be found within a CNN itself and does not require learning from a separate training dataset or manual design. An optimization objective in image restoration tasks is often defined as

(1) $$\min_{x}E\left(x;x_{0}\right)+R\left(x\right),$$

where x is the original image, $x_{0}$ is the corrupted image, $E(x;x_{0})$ is the data term which is the negative log of the likelihood and R(x) is the image prior term which is the negative log of the prior.

The traditional approach is to initialize *x* with some random noise, compute the gradient of the function with respect to *x* and pass through the image space until reaching a point of convergence. Thus, the optimization is evaluated in the image space. Unlike the conventional approach, the DIP approach [25] proposes performing a surjective g:θ↦x. In this way, we obtain

(2) $$\min_{\theta }E\left(g\left(\theta \right);x_{0}\right)+R\left(g\left(\theta \right)\right).$$

This equation is, in theory, equivalent to Equation (1). In this approach, the function *g* is initialized with random values of θ. The output from function *g* is then mapped to the image space and updated θ using gradient descent.

The novel approach gets rid of the prior term by selecting an appropriate value for *g*, and g(θ) can be defined as $f_{\theta }\left(z\right)$, where f is a deep convolutional network with the parameters θ and *z* is a fixed input. Then, Equation (2) is then reformulated as

(3) $$\min_{\theta }E\left(f_{\theta }\left(z\right);x_{0}\right).$$

Therefore, instead of searching for the answer in the image space, we now search for it in the space of the neural network’s parameters. Image statistics are captured by the structure of a convolutional image generator rather than by any previously learned capabilities.

### 2.2. Classical Single-Image Gap-Filling Methods

The performance of our DIP approach was compared with three other popular gap-filling methods: kriging interpolation [8], weighted linear regression (WLR) [14] and the direct sampling (DS) method [15].

Kriging interpolation, also known as Gaussian process regression [30], assumes that the distance or direction between the reference points reflects a spatial correlation that can be used to explain the change on the surface:

(4) $$\hat{Z}\left(s_{0}\right)=\sum_{i=1}^{N}\lambda_{i}Z\left(s_{i}\right),$$

where $Z\left(s_{i}\right)$ is the measured value at the location, $\lambda_{i}$ is the unknown weight for the measured value in the location, $s_{0}$ is forecast location and *N* is number of measurement values. When using the kriging method, the weights are based not only on the distance between measured points and forecast locations but also on the overall spatial location of the measured points. To use spatial location in the scales, the user must determine the amount of spatial autocorrelation. Thus, in ordinary kriging, the weight $\lambda_{i}=$ depends on the fitted model for the measured points, the distance to the forecast location and the spatial relationships between the measured values around the forecast location.

The multi-temporal recovery method based on WLR proposes that every single missing pixel can be recovered using a linear relationship calculated from locally similar pixels:

(5) $$Z\left(x_{i},t_{1},v\right)=aZ\left(x_{i},t_{2},v\right)+b,$$

where *a* and *b* are regression coefficients. After the selection of locally similar pixels, the regression coefficients *a* and *a* can be calculated using the weighted least squares method, and thus the pixels in the target image can be predicted using the WLR equation.

The DS method is a multipoint geostatistical method that fills in gaps by directly sampling either from the input image or from known parts of the target image. The basic idea is to find one pixel *y* in the non-corrupted part of the image whose neighborhood distance with a target pixel *x* is similar to that of the observed pixel *y*. In this study, a Euclidian distance is used for univariate simulation:

(6) $$d\left(N_{x},N_{y}\right)=\frac{1}{\eta }\sqrt{\sum_{i=1}^{n}{\left[Z\left(x_{i},t_{1},v\right)-Z\left(y_{i},t_{2},v\right)\right]}^{2}}\in \left[0,1\right],$$

where η is a normalization factor constrained to a distance between 0 and 1.

All these methods use learning-free techniques and can be applied as single-image methods along with the DIP approach. The simulation results of the single-image realization of these methods were taken from Yin et al. [17]. To make our results comparable with the numbers from the paper, we used the same data as in the above-mentioned article.

### 2.3. Study Area and Dataset

To investigate the reconstruction accuracy of deep image prior, we used a non-corrupted image from 24 July 2002 and a gap mask mimicking the SLC-off condition. For comparison of the gap-filling methods, Landsat-7 bands 1–4 were used in this study. The bands correspond to the red, green and blue portions of the visible spectrum and near-infrared spectral range. In addition, gap masks of different widths were overlaid on the original image to simulate different percentages of corrupted pixels, thus simulating different parts of the Landsat-7 scene that could be corrupted to different levels. The satellite data were obtained from USGS EarthExplorer (https://earthexplorer.usgs.gov/, accessed on 21 November 2022).

The territory covered by the investigated image is located in California, USA at around 37.97° N, 121.51° W and comprises an area of 12 km ${}^{2}$. The area is rather heterogeneous, consisting of vegetation cover, bare soil, open water and impervious surfaces. This region was chosen for the experiments in order to facilitate a comparison of the results from DIP with those from the classical gap-filling methods investigated in an article by Yin et al. [17].

### 2.4. Accuracy Evaluation

We evaluated the performance of the gap-filling simulations using classical metrics: the root mean squared error (RMSE) and $r^{2}$ score. The RMSE is a metric representing the square root of the average squared error computed for each pixel:

(7) $$\mathrm{RMSE}=\sqrt{\frac{\sum_{i=1}^{N}{\left(x_{i}-{\hat{x}}_{i}\right)}^{2}}{N}},$$

where *i* is a variable, *N* is the number of pixels, $x_{i}$ is a ground truth pixel and ${\hat{x}}_{i}$ is a reconstructed pixel. Lower values for the RMSE indicate more accurate simulations. However, the RMSE could be an inappropriate metric in the case of noisy data.

The second metric we used was the $r^{2}$ score or the coefficient of determination. It provides information about how well the model would replicate the observed outcomes based on the proportion of total variation of the outcomes explained by the model [31]. Assuming we have a model f(·) and true value $x_{i}$, the $R^{2}$ score between the predicted value $\hat{x}$ and the corresponding value *x* is defined as

(8) $$R^{2}\left(x,\hat{x}\right)=1-\frac{\sum_{i}{\left({\hat{x}}_{i}-x_{i}\right)}^{2}}{\sum_{i}{\left(x_{i}-\hat{x}\right)}^{2}}$$

$R^{2}$ shows the quality of model fitness, with higher values indicating better predictions. The best possible value of $r^{2}$ score is 1.0.

### 2.5. Experimental Setting

**Figure 1:**
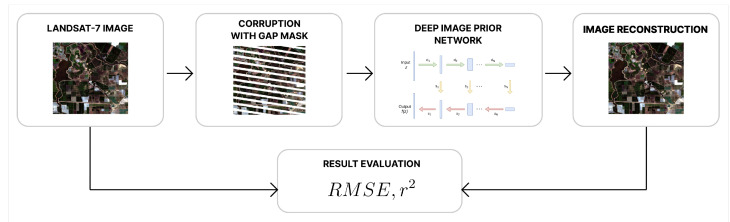
Outlines of the research.

The satellite image restoration was carried out in two different ways. First, we filled the gaps for each band separately. In this case, an input $x_{0}$ for the deep image prior network was a single band. The number of hidden (or corrupted) pixels was fixed at 55%. In the second approach, we stacked all four spectral bands and trained the network on the obtained composite. We used the composite to estimate the influence of the number of corrupted pixels on the ability of deep image prior to fill the values. We conducted five simulations with image corruption levels of 3%, 6%, 15%, 35% and 55% of the whole area. The outlines of the research are presented in Figure 1.

For our convolutional network, we used the “hourglass” (also known as “decoder-encoder”) architecture that was used in the original deep image prior paper [25]. The main part of the code regarding the CNN training was acquired from the publicly available implementation (https://github.com/DmitryUlyanov/deep-image-prior, accessed on 21 November 2022). We trained our deep image prior model for 1500 epochs using the Adam solver [32] with a batch size of 1. We used LeakyReLU [33] as a nonlinearity. As an upsampling operation, nearest neighbor upsampling was used. The input vector *z* was filled with uniform noise between 0 and 0.1. The architecture and hyperparameters details are presented in Appendix A. All the experiments were conducted using the Python programming language and Pytorch open source machine learning framework.

## 3. Results and Discussion

This section provides the results of the experiments and a comparison of DIP with classical single-image gap-filling methods. All the experiments provided in this paper are available at a GitHub repository (https://github.com/petrovskaia/deep-image-prior-landsat, accessed on on 21 November 2022).

The quantitative comparison of the separate bands of given data for our method is given in Table 1, showing a quantitative advantage of the proposed approach compared with classical gap-filling methods. Here, $r^{2}$ was significantly higher for each reconstructed band. The lowest-performing restoration made by the DIP approach reached 0.812 (Band 1), while the best value for the classical approaches for this band was 0.685, achieved by the DS method. The results show the capability of deep image prior in fitting the model. This result also demonstrates that deep image prior was able to identify the patterns in the satellite image remarkably better than the other methods considered. However, regarding the RMSE, the DIP approach had noticeably higher values.

**Figure 2:**
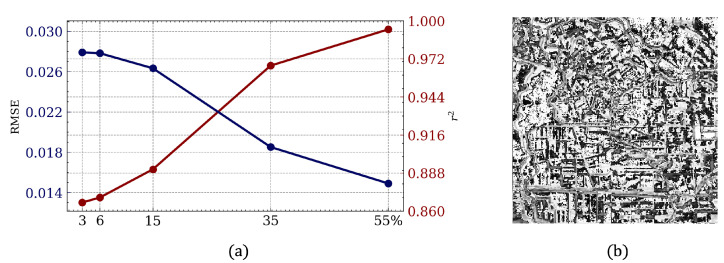
Simulation results. (**a**) Comparative statistics. X-axis: percentage of corrupt pixels. Y-axis (left): root mean squared error (RMSE) between simulated and real pixel values. Y-axis (right): corresponding coefficient of determination ($r^{2}$). (**b**) Pixel-wise similarity visualization for case with 55% hidden pixels (worst case scenario). White shows higher level of similarity, while dark gray represents dissimilarity.

The explanation for a mismatch in performance measured by the RMSE and $r^{2}$ is connected to the process of DIP training and the procedure of filling the gaps. Hence, DIP does not simply fill in only the missing values; it reconstructs the entire image using the non-corrupted parts as guidelines from the training. This peculiarity of the training process allows DIP to achieve impressive results with regard to the correlation between the prediction and ground truth. However, at the same time, the non-corrupted parts of the initial image become slightly degraded after the restoration process. This can be clearly seen in Figure 2b, which shows the similarity between the original image (ground truth) and reconstruction from the 55% hidden pixels scenario. The figure is very heterogeneous, and the parts that were corrupted in the original image cannot be distinguished from this figure.

As for the training settings, we found that using a composite of four bands gave better results than reconstructing each band separately. We conducted this experiment with 55% corrupted values, the highest possible level of corruption in the Landsat-7 scenes. The average $r^{2}$ for separate training was 0.842, and the $r^{2}$ for the composite was −0.880. The performance with respect to the RMSE was also marginally better for the composite training than for separate bands (0.030 vs. 0.034, respectively).

In order to show that the proposed approach is applicable to different degrees of image corruption, we provide experiments with different gap masks. The results are presented in Figure 2a. The performance of DIP steadily decreased with the percentage of corrupted pixels. The best results were achieved for the image with 3% of it covered with a mask (−0.13 for RMSE and 0.976 for $r^{2}$). Overall, deep image prior was able to handle both small and large numbers of hidden pixels successfully.

**Figure 3:**
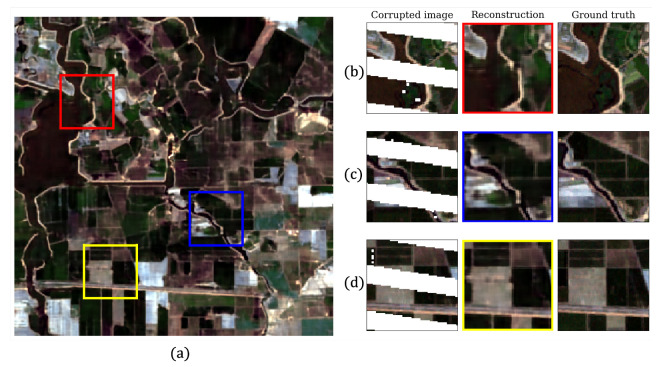
Comparison of DIP reconstruction (55% hidden pixels), image with gap mask and original image for three regions. (**a**) Result of reconstruction. (**b**–**d**) Parts of image overlapped with gap mask (corrupted image), corresponding parts of reconstructed image and parts of initial image (ground truth), respectively.

In Figure 3, we present a qualitative visual comparison of the reconstructed image with the original image and the image covered by the gap mask. This comparison was made for the most challenging case with the most considerable amount of hidden pixels (55%). When analyzing particular image parts, we found the gap-filling result to be entirely accurate, except for minor inaccuracies.

## 4. Conclusions

This work presented an application of the deep image prior deep learning approach to the restoration of corrupted Landsat-7 imagery. This paper demonstrated the superior capability of our approach over traditional single-image methods in learning the spatial patterns in the image. In addition, this paper provided an examination of DIP specificities in the process of satellite image reconstruction.

The proposed method uses only the corrupted image in the restoration process and is beneficial for areas where multi-temporal snapshots may be unavailable due to reasons such as cloud cover or instrument failure. This approach could be further extended to problems with irregular artifacts on satellite images, such as the removal of clouds, which could further enhance the usability of remote sensing imagery. The usage of the proposed approach will expand the possibilities for a wide variety of agricultural studies and applications.

## Figures and Tables

**Table 1:** Comparison of performance of different popular gap-filling methods [17] and deep image prior. Bold text highlights the best value for a band. The DIP approach outperformed the other methods according to $r^{2}$ score for all bands.

	RMSE				*r* ^2^			
Method	Band 1	Band 2	Band 3	Band 4	Band 1	Band 2	Band 3	Band 4
Kriging	0.010	0.015	0.023	0.063	0.610	0.627	0.728	0.690
WLR	0.010	0.014	0.023	0.055	0.622	0.694	0.742	0.765
DS	**0.009**	**0.012**	**0.020**	**0.052**	0.685	0.755	0.792	0.780
DIP (ours)	0.020	0.024	0.043	**0.052**	**0.812**	**0.853**	**0.874**	**0.832**

**Figure A1:**
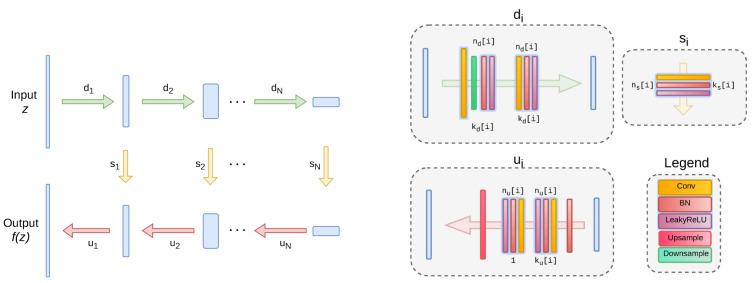
The architechture used for image restoration, where $n_{u}\left[i\right],n_{d}\left[i\right]andn_{s}\left[i\right]$ correspond to the number of filters at depth *i* for the upsampling, downsampling and skip connections, respectively. The values $k_{u}\left[i\right],k_{d}\left[i\right]andk_{s}\left[i\right]$ correspond to the respective kernel sizes. The image were taken from the suplemantary materials in [25].

## Data Availability

All the data and experiments from the paper are available at the following Github repository: https://github.com/petrovskaia/deep-image-prior-landsat, accessed on 21 November 2022.

## Abbreviations

    The following abbreviations are used in this manuscript:

DIP	Deep image prior
RMSE	Root mean squared error
DS	Direct sampling
WLR	Weighted linear regression

## Appendix A. Architecture Details

The architecture and hyperparameters were chosen by following those in [25]. We used the encoder-decoder architecture with four skip connection layers. As a downsampling technique, we used strides implemented within convolution modules. As an upsampling operation, we used nearest neighbor upsampling. The input for the network was meshgrid, initialized with $z\in R^{2\times W\times H}$ using np.meshgrid. The other details of the architecture are provided below. The explanation is in Figure A1, taken from the supplementary materials of [25].

$z\in R^{4\times W\times H}∼U\left(0,\frac{1}{10}\right)$
$n_{u}=n_{d}=$[128, 128, 128, 128, 128]
$k_{u}=k_{d}=$[3, 3, 3, 3, 3]
$n_{s}=$[128, 128, 128, 128, 128]
$k_{s}=$[1, 1, 1, 1, 1]
$\sigma_{p}=\frac{1}{10}$
num_iter = 1500
LR = 0.01
upsampling = nearest
